# Identification of a Novel *Calotropis procera* Protein That Can Suppress Tumor Growth in Breast Cancer through the Suppression of NF-κB Pathway

**DOI:** 10.1371/journal.pone.0048514

**Published:** 2012-12-20

**Authors:** Ramar Perumal Samy, Peramaiyan Rajendran, Feng Li, Narayana Moorthy Anandi, Bradley G. Stiles, Savarimuthu Ignacimuthu, Gautam Sethi, Vincent T. K. Chow

**Affiliations:** 1 Infectious Diseases Programme, Department of Microbiology, Yong Loo Lin School of Medicine, National University Health System, National University of Singapore, Singapore, Singapore; 2 Department of Pharmacology, Clinical Research Centre, Yong Loo Lin School of Medicine, National University Health System, National University of Singapore, Singapore, Singapore; 3 Entomology Research Institute, Loyola College, Chennai, Tamil Nadu, India; 4 Integrated Toxicology Division, United States Army Medical Research Institute of Infectious Diseases, Fort Detrick, Maryland, United States of Amercia; 5 Wilson College, Chambersburg, Pennsylvania, United States of America; University of Michigan School of Medicine, United States of America

## Abstract

Breast cancer is the most common cancer among women. To date, improvements in hormonal and cytotoxic therapies have not yet led to a sustained remission or cure. In the present study, we investigated the *in vitro* and *in vivo* antitumor activities of a novel *Calotropis procera* protein (CP-P) isolated from root bark. CP-P protein inhibited the proliferation and induced apoptosis of breast cancer cells through the suppression of nuclear factor kappaB (NF-kB) activation. CP-P, when administered individually or in combination with cyclophosphamide (CYC, 0.2 mg/kg) to rats with 7, 12-dimethyl benz(a)anthracene (DMBA)-induced breast cancer decreased tumor volume significantly without affecting the body weight. To elucidate the anticancer mechanism of CP-P, antioxidant activities such as superoxide dismutase (SOD), catalase (CAT), glutathione-s-transferase (GST) and non-enzymatic antioxidant - reduced glutathione (GSH), vitamin E and C generation in the breast were analyzed by various assays. SOD, CAT, GST, GSH, vitamin E and C levels were high in combination-treated groups (CP-P+CYC) versus the CYC alone-treated groups. Also, the combination was more effective in down-regulating the expression of NF-kB-regulated gene products (cyclin D1 and Bcl-2) in breast tumor tissues. Our findings indicate that CP-P possesses significant antitumor activity comparable to a commonly used anticancer drug, cyclophosphamide, and may form the basis of a novel therapy for breast cancer.

## Introduction

Breast cancer (BC) is one of the leading causes of death in women worldwide [1] and ranked second after cervix and lung cancer. Recently reported known risk factors, such as mammographic density [2], [3], [4], [5], genetic modifications of interleukin-18 (IL-18) [6], tumor protein 53 (p53), breast cancer 1 (BRCA1) and breast cancer 2 (BRCA2) genes [7], [8], [9] account for approximately 30% of breast cancer cases. However, exposures to environmental chemicals like polycyclic aromatic hydrocarbons (PAHs), obesity [10], and other life style-related factors evidently play a pivotal role in this increased incidence [11], [12]. Although breast cancer incidence has increased over the past four decades, mortality has remained constant, due to the vast improvement in detection and treatment options. However, there is a relative lack of effective therapies for advanced-stage metastatic disease. Thus, there is an urgent need to develop novel therapeutic agents against advanced breast cancer, which are expected to be safe and effective in this age class [1], [13].

Several agents derived from natural sources exhibit anti-cancer properties without considerable adverse effects and play a vital role for developing new drugs [14], [15], [16], [17], [18]. In addition, epidemiological data suggest that consumption of plant foods or natural products (47.2%) and botanical supplements (47.5%) [19], which contain high levels of antioxidants, might slow down or prevent the appearance of cancer [20]. *Calotropis procera* (L.) R.Br. belongs to the family Asclepidaceae and is well known for its various medicinal properties [21]. Different parts of leaves, roots, flowers and latex from this plant are used in several Ayurvedic systems of medicinal preparations [22]. The leaves of *C. procera* are effective in treating migraines [23]. Leaves and stem bark extracts of *C. procera* are widely used in Asian and West African traditional therapy for dermatological and bronchial infections [24]. In addition, the aqueous stem bark extract possesses cough-suppresant activity against bronchial irritation caused by organic solvent ammonia in Guinea pigs [25]. The decoction of the aerial part of *C. procera* is commonly used in Saudi Arabian traditional medicine for treating various diseases including fever, joint pain, muscular spasm and constipation. Ethanol extract of the plant has been tested on laboratory animals for its antipyretic, analgesic, antibacterial, anti-inflammatory, purgative [26], and muscle relaxant activities [27]. The stem bark fractions of n-hexane, 1-butanol, ethyl acetate, chloroform and water showed significant anti-inflammatory activity at 200 and 400 mg/kg [28]. Additionally, the root bark of *C. procera* has been found to produce marked improvement in cases of diarrhoea and dysentery [29]. The effect of an ethanolic extract of *C. procera* roots has been studied in albino rats to explore its antifertility and hormonal activities [30]. Effects of ethanol and aqueous extract from *C. procera* roots have also been analyzed upon the oestrous cycle in rats [31].

The milky latex from this plant has been used in traditional medicine to cure skin infections, poison, ulcer, enlargement of spleen, liver, abdominal glands, colic piles, worms and different inflammatory diseases [32]. Based on its traditional use, this latex was selected for evaluation of its wound healing potential [24]. The clinical and pathological effects of *C. procera* latex have been well investigated [33]. It showed mild toxic effects on heart, liver and kidneys; that included multi-focal coagulation necrosis of cardiac fibers and vacuolized hepatocytes [34]. Different proteins like laticifer [35], and osmotin from the latex of *C. procera* reportedly exhibit potent anti-fungal [36], antimycoplasmal [37], anti-inflammatory [38], [39], insecticidal [40], larvicidal [41], [42], antioxidant and radical scavenging activities [43], and anticancer [44] properties. However, to date there is no literature available for the anticancer effects of *C. procera* root bark. Because *Calotropis procera* protein (CP-P) exhibits anti-inflammatory, anti-oxidant, and antipyretic effects, we postulated that CP-P may mediate its effects by modulating the NF-κB activation pathway. The latter has been closely linked to inflammation, tumorigenesis, proliferation, invasion, angiogenesis, and metastasis. The NF-κB pathway is activated in response to various inflammatory agents, carcinogens, tumor promoters, and growth factors. Hence, in the present study we aim to investigate whether the antiproliferative/pro-apoptotic *in vivo* effects of CP-P on 7, 12-dimethyl benz(a)anthracene (DMBA) induced breast cancer in rat models are mediated through the suppression of NF-κB signaling pathway. Our results indicate that CP-P inhibited the NF-κB activation pathway and the expression of various antiapoptotic and proliferative gene products, subsequently inducing apoptosis and suppression of tumor growth in a DMBA-induced breast cancer model.

## Results

### Identification of novel antitumor protein

**Figure 1:**
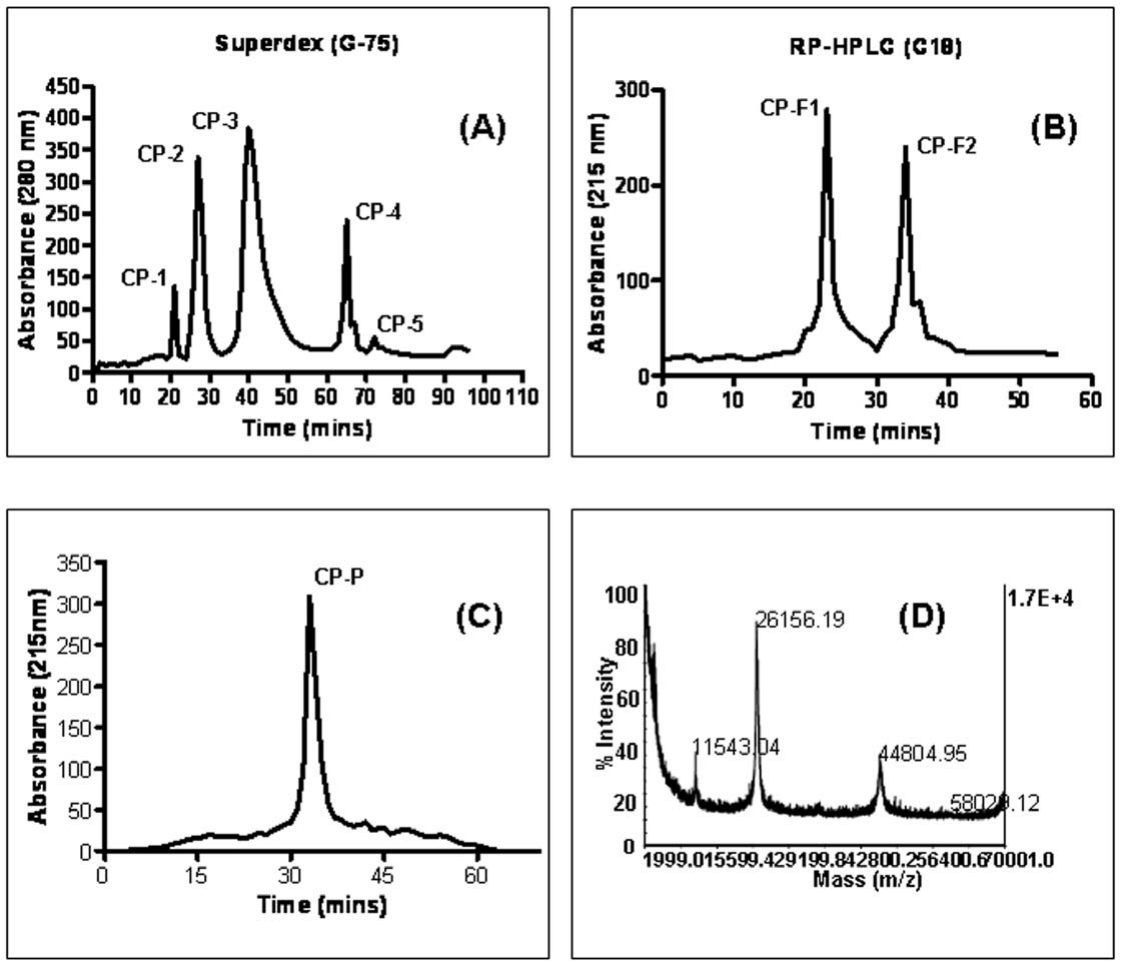
Purification and characterization of anticancer protein CP-P. (A) The clear supernatant of *Calotropis procera* root-bark was separated by gel-filtration (Superdex G-75) chromatography into five major peaks CP-1–CP-5, (B) the most active peak (CP-3) was further separated by reverse-phase high performance liquid chromatography (RP-HPLC) by using a C18 column which produced two peaks namely CP-F1 and CP-F2, (C) the active fraction (CP-F1) was resolved by a C8 column and gave a single peak named *Calotropis procera* protein “CP-P”, (D) CP-P mass was analyzed by a perspective biosystem matrix-assisted laser desorption ionization-time of flight (MALDI-TOF/MS).

A clear supernatant of *C. procera* extract was separated by gel-filtration chromatography on Superdex G-75 column, yielding five major peaks CP-1–CP-5 (Figure 1A). Fraction CP-3, showing the highest recovery of total protein and antitumor activity, was further fractionated by RP-HPLC on a C18 column and resolved into two protein fractions, named CP-F1 and CP-F2 (Figure 1B). CP-F1 was further applied to a HPLC reverse-phase (C8) column and resolved into a single isolated protein CP-P (Figures 1C). The mass of the pure protein was determined by MALDI-TOF/MS (molecular weight 26156.19 kDa) (Figure 1D). The search program MS-Fit and was used for searches in the database NCBI BLAST and MSDB protein databases using Mascot tool. The Mascot search results revealed that the protein (CP-P) matched a previously reported apolipoprotein A-I protein (more details refer to supplementary files in Table S1, S2).

### *In vitro* cytotoxic effect of CP-P in breast cancer cells

**Figure 2:**
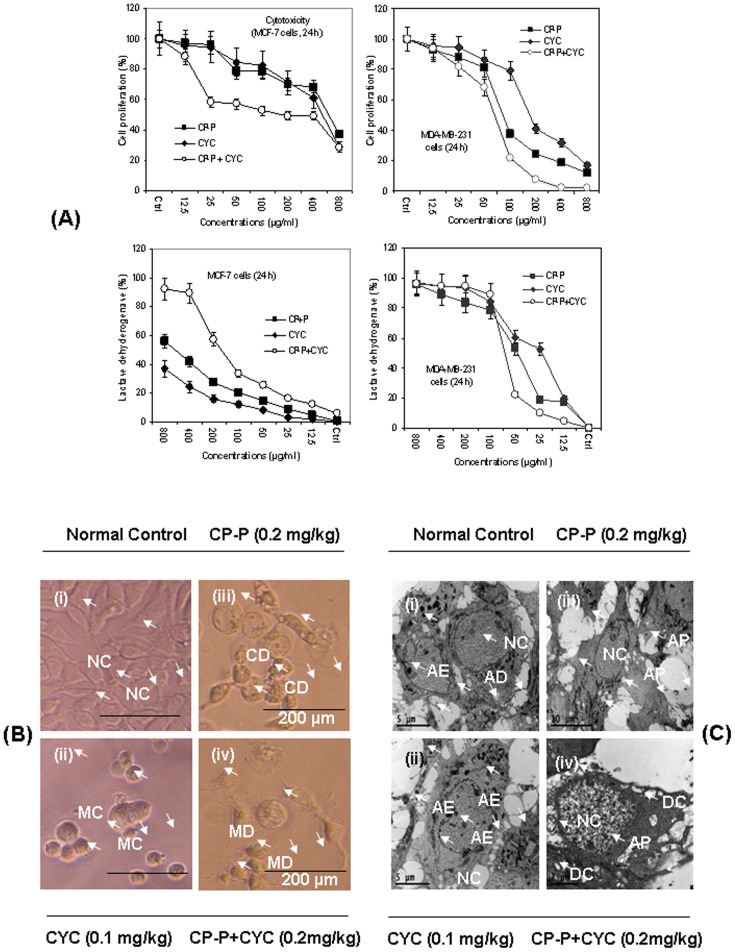
The antiproliferative effect of CP-P evaluated against MCF-7 and MDA-MB-231 human breast cancer cells. (A) *In vitro* cytotoxicity of CP-P, with or without CYC, upon MCF-7 and MDA-MB-231 cells at different doses (800–12.5 µg/ml). The CP-P alone, or when combined with CYC, leads to significant (*P<0.01) reduction in cell proliferation even at 12.5 and 50 µg/ml doses versus untreated control. The MCF-7 cells were more sensitive against the combination treatment. Values are mean ± SD of three replicates. Untreated MCF-7 cells served as a control, CP-P treatment evidenced cell death (CD) at 100 µg/ml concentrations on MDA-MB-231 cells. The CP-P alone, or in combination with CYC, caused the most significant (*P<0.01) leakage of LDH. The combination treatment induced more cell-death and lytic effects in both cell lines than CP-P or CYC alone. (B) Light micrographs showing the MCF-7 cell morphology before and after treatment. (i) There was no effect on untreated MCF-7 cells as a control, (ii) protein (CP-P) or drug (CYC) alone treated cells induced severe cell membrane damage and inhibited MCF-7 cell growth, (iv) We also observed that MCF-7 cells treated with CP-P in combination with CYC showed remarkable morphological changes like cell shrinkage, formation of membrane blebs, nuclear and cytoplasmic condensation when observed under microscope (Symbol denotes: NC-Normal Control, MC-morphological changes, CD-Cell Death, MD - membrane damage). (C) Electron micrographs of MCF-7 cells exposed to CP-P alone, or with CYC, showing the cellular and morphological changes. The transmission electron microscopy (TEM) analysis was performed on MCF-7 human breast cancer cells exposed to the CP-P alone, or with CYC treatment to observe morphological changes. Electron micrographs of MCF-7 cells, (i) untreated control MCF-7 cells which showed normal morphology. (ii) *Calotropis procera* protein (CP-P) treatment exhibited cell death at 100 µg/ml concentrations, a portion of the nucleus dismantled within the cytoplasm and a large number of vacuoles were clearly visible in proteins treated cells. (iii) CYC alone treatment caused blebbing as revealed by the presence of large vacuoles on MCF-7 treated cells. (iv) These results further confirmed that the combination treatment of CP-P+CYC induce MCF-7 cells death at 12.5 µg/ml doses. MCF-7 cells treated with CP-P+CYC showed apoptotic morphology including chromatin condensation into dense granules or blocks. The cells showed loss of microvilli and blebbing of cell membrane which are characteristics of cells undergoing apoptosis.

We next investigated the effects of CP-P alone, or in combination with CYC, on inhibition of growth and proliferation of human MCF-7 and MDA-MB-231 breast cancer cells using MTT and LDH bioassays. In single and combination treatments, CP-P and CP-P+CYC inhibited cell growth and decreased cell survival through induction of cell death in a dose-dependent manner. The data revealed that MDA-MB-231 cells were more sensitive to combination, versus single, treatment regimen (P<0.01) (Figure 2A). The opposite relationship between the LDH and MTT responses adds credence to the data accuracy. In the LDH assay, as the concentration of the CP-P+CYC or CP-P treatment increased, MCF-7 cells became progressively more cytotoxic, leading to a greater absorbance reading in the LDH assay and decreased absorbance in the MTT assay with a concurrent decrease in the percentage of viable cells (Figure 2A). In addition, the microscopic images clearly supported the findings, as no changes were observed in untreated cells. In contrast, the CP-P-exposed MCF-7 cells became necrotic after 24 h (Figure 2B). The CYC-treated cells became round in shape. However, the combination treatment completely damaged the breast cancer cells. The ultra-structural examination demonstrated that the untreated cells showed normal architecture (Figure 2C), MCF-7 cells exposed to CP-P showed cellular and morphological changes that included cell death (Figure 2C). Standard drug CYC alone also showed adverse effects on MCF-7 cells. Overall these observations further confirmed that the combination treatment of CP-P+CYC can induce MCF-7 cells death by apoptosis. MCF-7 cells treated with CP-P+CYC showed apoptotic morphology including chromatin condensation into dense granules or blocks. The cells showed loss of microvilli and blebbing of cell membrane which were characteristics of cells undergoing apoptosis (Figure 2C).

### CP-P induces apoptosis of breast cancer cells

**Figure 3:**
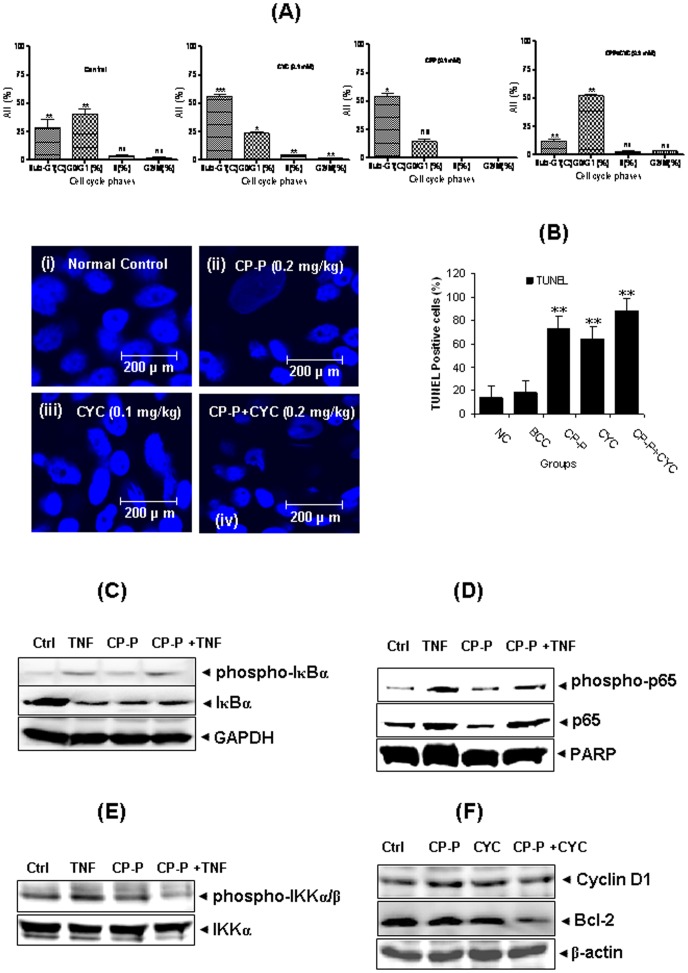
The number of apoptotic cells in breast tumor sections was detected by TUNEL assay. (**A**) The apoptotic effect of CP-P alone or in a combination of CYC treatment was analyzed by flow cytometry to compare the various cell cycle phases. The CP-P alone, or in combination with CYC, induced promising apoptotic effects. As a result, fewer MCF-7 cells accumulated in the Sub-G1 phase compared to the control. (**B**) TUNEL positive cells were quantified, revealing that apoptotic cells in breast tumors increased significantly in CP-P or CYC drug alone, or in combination, groups versus untreated cells. CP-P and CYC drug-treated groups were compared to breast cancer control groups. The combination treatment induced more positive cells than others. Values are mean ± S.D. (n = 3) replicates, **P<0.01 values indicate significance to control. (**C**) CP-P inhibits TNF-α -induced nuclear degradation/phosphorylation of IκBα. MCF-7 cells were either untreated or pretreated with 50 µg/ml CP-P for 6 h at 37°C and then treated with 1 nM TNF-α for 30 min. Cytoplasmic extracts were prepared and analyzed by Western blotting using antibodies against anti- IκBα and phospho-specific IκBα. The same membrane was reblotted with GAPDH antibody to verify equal loading. The results shown are representative of two independent experiments. (**D**) CP-P inhibits TNF-α-induced nuclear phosphorylation/translocation of p65. MCF-7 cells were either untreated or pretreated with 50 µg/ml CP-P for 6 h at 37°C and then incubated with 1 nM TNF-α for 30 min. Nuclear extracts were prepared and analyzed by Western blotting using antibodies against anti-p65 and phospho-specific p65. The same membrane was reblotted with PARP antibody to verify equal loading. The results shown are representative of two independent experiments. (**E**) Effect of CP-P on TNF-induced phosphorylation of IKK-α and IKK-β. MCF-7 cells were incubated with 50 µg/ml CP-P for 6 h at 37°C and then treated with 1 nM TNF-α for 15 min. Whole cell extracts were prepared and analyzed by Western blot analysis using anti-phospho-specific IKK-α/β antibody. The same membrane was reblotted with anti-IKK-α antibody to verify equal loading. (**F**) CP-P inhibits TNF-α -induced expression of NF-κB-dependent proliferative and antiapoptotic proteins. MCF-7 cells were incubated with 50 µg/ml CP-P for 6 h at 37°C and then treated with 1 nM TNF-α for 24 h. Whole-cell extracts were prepared and analyzed by Western blot using the indicated antibodies (Cyclin D1 and Bcl-2). The same membrane was reblotted with β-actin antibody to verify equal loading. Results are representative of two independent experiments.

We also investigated the apoptotic potential of CP-P apoptotic potential in breast cancer cells. Cell cycle analysis was used to analyze the distribution of MCF-7 cells in different phases of the cell cycle following exposure to CP-P alone, or in combination with CYC, after 24 h. We found that the apoptotic phase (Sub-G1 phase) was significantly increased in cells treated with CP-P+CYC after 24 h. In addition, CP-P+CYC induced apoptotic effects were statistically significant (**p value <0.05) after 24 h (Figure 3A). However, the accumulation of breast cancer cells in the Sub-G1 (apoptotic) phase increased only slightly after treatment with CP-P and CYC at 24 h. The TUNEL assay showed the typical hallmark morphological changes associated with apoptosis which includes chromatin condensation, protein breakdown and DNA fragmentation in the treated cells. The results showed a significant amount of apoptotic cell death in the MCF-7 cells when compared with the normal cells (Figure 3B, i–iv). Extensive cell death was observed in proliferating human breast cancer cells after treatment with CP-P, and in combination with CYC, through cytotoxic necrosis and apoptosis.

### CP-P inhibits TNF-α -dependent IκBα degradation and phosphorylation

Because IκBα degradation is essential for the activation of NF-κB [46], we determined whether inhibition of TNF-α -induced NF-κB activation by CP-P was due to the inhibition of IκBα degradation. We found that TNF-α -induced IκBα degradation in control MCF-7 cells, but in CP-P-pretreated cells TNF-α had little effect on IκBα degradation (Figure 3C). To determine whether the inhibition of TNF-α-induced IκBα degradation was due to suppression in IκBα phosphorylation, we pretreated cells with CP-P, exposed them to TNF-α, and then determined IκBα phosphorylation levels in the cytoplasm by Western blot. TNF-α induced IκBα phosphorylation while CP-P almost completely suppressed it (Figure 3C, middle panel).

### CP-P inhibits tumor necrosis factor (TNF-alpha)-induced phosphorylation/translocation of p65

TNF-α induces the phosphorylation of p65, which is required for transcriptional activity [45]. After phosphorylation, the p65 subunit is translocated to the nucleus. In the nuclear fraction from the TNF-α -treated MCF-7 cells, there was a time-dependent increase in the phosphorylated form of p65 that was suppressed by CP-P. Western blot analysis also revealed that TNF-α induced nuclear translocation of p65; and CP-P clearly suppressed its translocation to the nucleus (Figure 3D, middle panel). The same membrane was reblotted with PARP antibody to verify equal loading. The results shown are representative of two independent experiments

### CP-P inhibits TNF-α -induced phosphorylation of IKK in MCF-7 cells

TNF-α has been reported to activate TAK1 leading to the phosphorylation of both IKK-α and IKK-β [47]. Whether CP-P can modulate phosphorylation of IKK-α and IKK-β in breast cancer cells was investigated. The results shown in Figure 3E indicate that TNF-α induced the phosphorylation of both IKK-α and IKK-β, and CP-P abolished TNF-α induced IKK activation.

### CP-P represses the expression of TNF-α -induced NF-κB-dependent gene products involved in proliferation and survival

We next investigated whether CP-P can modulate the expression of NF-κB -regulated gene products involved in the proliferation and survival of tumor cells. Pretreatment with CP-P inhibited TNF-α induced expression of cyclin D1 and Bcl-2 in MCF-7 cells as observed by western blot analysis (Figure 3F). The suppression of cyclin D1 and Bcl-2 may explain anti-proliferative and pro-apoptotic activities of CP-P as described above.

### *In vivo* antitumor activity of CP-P in rats

**Figure 4:**
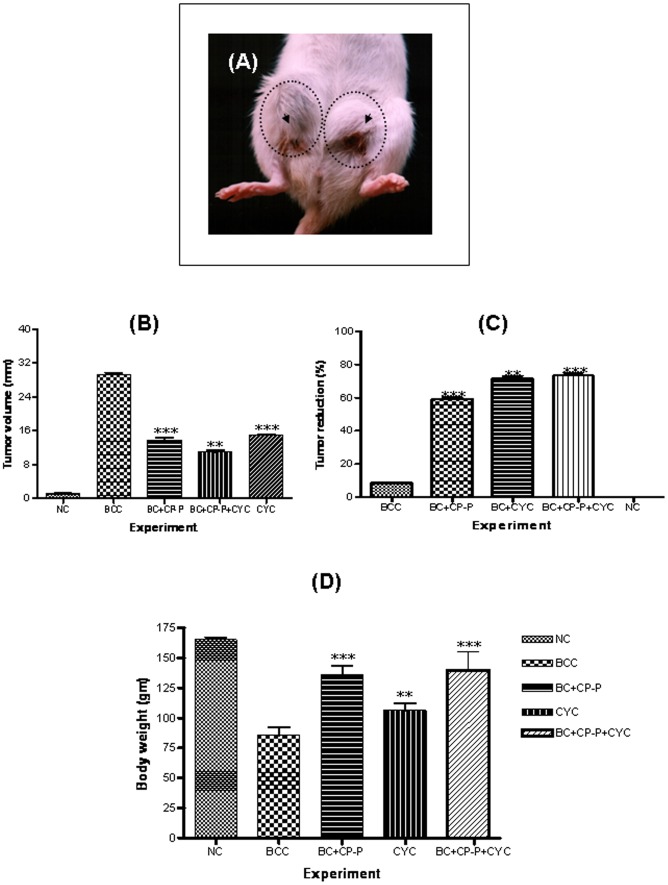
Antitumor effect of CP-P (0.1 mg/kg, oral administration)+CYC (0.1 mg/kg, intra peritoneal injection) treated on DMBA-induced mammary tumors in rats after 110 days. (A) Microscopic images showed that the DMBA-induced breast tumor development after 90 days on either side of the mammary gland are marked by a circle and an arrow pointed out. (B) Breast tumor volume was measured and calculated as 30 millimeter in diameter in breast cancer control panel and the tumor incidence was compared within the groups. (C) Percentage reduction of tumor volume was measured after 20 days treatment of CP-P (0.1 mg/kg, oral administration)+CYC (0.1 mg/kg, intra peritoneal injection). (D) Body weight loss before and after the treatment. Body weight changes of different treatments compared within the five different groups (Symbol denotes: NC-Normal Control, BBC - Breast Cancer Control).

Each breast cancer control (BCC), rat received 1 mg of DMBA that induced 100% multiplicity of mammary carcinomas during a median latent period from 3 to 13 weeks (Figure 4A). The anti-tumor effects of CP-P on DMBA-induced mammary carcinogenesis are shown. CP-P (0.2 mg/kg) when orally administered for 20 days suppressed DMBA-induced mammary gland carcinogenesis in rats. In contrast, treatment with CP-P+CYC had significant effects on mammary tumorigenesis after 3 weeks. Moreover, in addition to the *in vivo* study showing a significant decrease in tumor volume (Figure 4B), a significant increase in the life span of rats was observed as compared to untreated group, the life span of CP-P treated animals increased by 53.4% (0.2 mg/kg) and 73.9% (CP-P 0.1 mg/kg)+CYC (0.1 mg/kg) effectively inhibited DMBA-induced tumors in rats (Figure 4C). The tumor latency period was calculated from the time at which 50% of the tumors had appeared in the breast tumor control group. The CP-P alone or in combination of CYC at 0.2 mg/kg, body weight doses exhibited antitumor activity as revealed by the significant increase in mean survival time and percent increase in life span of tumor-bearing rats. However, the CYC treatment caused a significant decrease in the body weight below the normal indicating toxicity (Figure 4D). There was a lesser mammary tumor incidence in mice treated with CYC alone versus BBC (p<0.05).

### Histopathological analysis of CP-P treated tumor tissues

**Figure 5:**
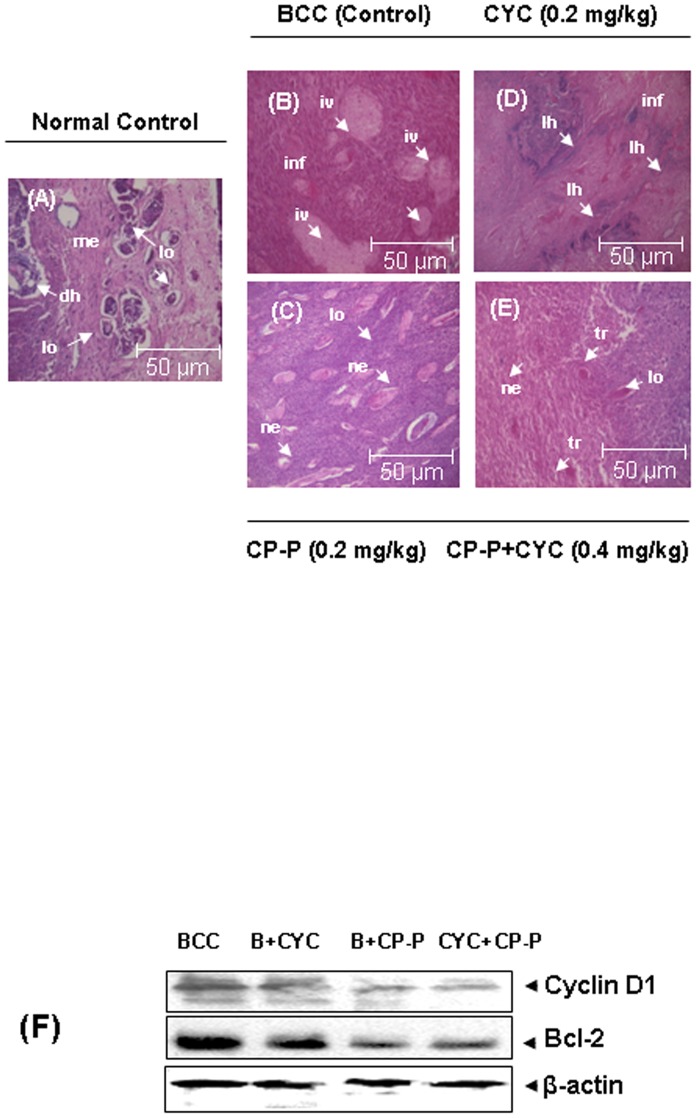
Histopathological examination of mammary tumor tissues. (A) Normal histology of breast tissue consisting lobules (l), myoepithelial (me), ductal hyperplasia (dh) with basement membrane. (B) Carcinoma of the rat mammary gland with extensive solid areas in small cluster. (C) Highly invasive (iv) tumor cells surrounding residual area and expanding into the adjacent stroma. Inhibitory potential of CP-P alone or in combination with CYC on DMBA-induced mammary tumors. (D) CYC inhibited the formation of lobular hyperplasia (lh) that is precursor to invasive disease. (E) The combination of CP-P+CYC treatment significantly reduced the number of inflammatory cells, hyperplasia and dysplasia respectively (Magnification ×20). Symbol denotes: lo-lobules, me-myoepithelial, dh-ductal hyperplasia, iv-invasive solid areas, lh-lobular hyperplasia, ne- neovascularization. (F) CP-P inhibits expression of NF-κB-regulated gene products in breast cancer tissue samples. Western blot analysis showed the inhibition of the expression of Cyclin D1 and Bcl-2 by CP-P in whole cell extracts obtained from tumor tissues.

Histopathological examination revealed a clear architecture of normal mammary glands (Figures 5A), whereas the breast cancer-bearing rats showed severe tumor tissue necrosis and lesions occurring after 13 weeks (90 days) of DMBA treatment (Figure 5B). Thus, the CP-P individually treated group III rats showed a reduction in the number of inflammatory cells (Figure 5C). Whereas, the CYC alone treated rat group V also showed faster epithelialization than the BCC treated rats (group II). In addition, we found necrotic (ne), infiltration (i) of tissues by the chemotherapeutic (CYC) effects on rats (Figure 5D). On the other hand, histological evidence clearly proved the antitumor potential in combination treated rats (groups IV) after 20 days (Figure 5E). In addition, the images also showed that there was accelerated epithelialization, tremendous reduction of infiltration and absence of necrotic effects when compared to that of normal control rats group I NF-κB is known to regulate the expression of number of proteins, including those involved in proliferation (cyclin D1), and survival (Bcl-2). Whether CP-P can modulate the expression of these NF-κB-regulated gene products in tumor tissues, was examined by western blot analysis. We found that treatment with CP-P was effective in down regulating the expression of cyclin D1 and Bcl-2 in tumor tissues (Figure 5F).

### Effect of CP-P on superoxide dismutase radical scavenging activity (SOD)

Antioxidant enzyme levels were decreased in the BCC rats after 13 weeks (group II). The CP-P alone treated group (0.2 mg/kg) (III) showed significantly (P<0.01*) elevated levels of SOD following 20 days of oral treatment. The combination-treated group (IV) exerted tremendous changes in SOD levels. Within the CYC group (V), SOD led to pronounced level of increment as compared to the NC (group I).

The GST levels were higher for the CP-P (group III) individually-treated groups than the CP-P+CYC treatment group (V) (Tables 1). The GST levels were significantly reduced in BCC groups (group II), whereas CP-P-treated groups showed higher levels of enzymatic antioxidant levels. In the CYC alone-treated group IV, GST levels were significantly increased at 0.2 mg/kg after 3 weeks. The enzymatic levels were not changed in the control group (group I). The BCC rats (group II) showed significant enzyme reduction after 13 weeks. However, CP-P individually-treated groups (III) versus the CP-P+CYC group (V) induced higher catalytic activity and suppressed the DMBA action. The CYC alone-treated group (IV) showed higher levels of CAT activity due to the presence of mutagenic chemicals as a side effect at 0.2 mg/kg dose.

**Table 1:** The activities of antioxidant enzymes levels in breast tissue of control and experimental animals.

Biochemical parameters	Group INC	Group IIBCC	Group IIIBC+CP-P	Group IVBC+CYC	Group VBC+CP-P+CYC
Superoxide dismutase^1^	4.16±0.51	1.60±0.27 a*	2.75±0.81 b*	2.80±0.28 b*	4.00±0.72 a§ b*
Catalase^2^	48.85±0.51	29.66±2.05 a*	31.33±2.27 b§	36.65±3.22 b*	46.21±3.2 a^NS^ b*
Glutathione-s-transferase^3^	1.33±0.13	0.97±0.17 a*	1.03±0.21 b§ *	1.10±0.11 b*	1.29±0.14 a^NS^ b*
Reduced glutathione^4^	3.08±0.34	1.83±0.27 a*	2.08±0.23 b^NS^	2.61±0.28 b*	2.98±0.14 a^NS^ b*
Vitamin C^5^	1.58±0.51	0.78±0.09 a*	0.91±0.15 b*	0.96±0.076 b*	1.47±0.13 a^NS^ b*
Vitamin E^6^	18.20±1.713	9.14±1.96 a*	12.06±1.02 b*	12.22±1.02 b*	16.83±1.16 a§b*

Values are mean ± SD (n = 3 replicates)

* P<0.001,

≠ P<0.01,

§ P<0.05,

NS - Not Significant (with respect to control), Abbreviations: NC - normal control, BCC - breast cancer control, BC - breast cancer, CYC - cyclophosphamide, CP-P - *Calotropis procera* protein, (a) - Group II and IV compared between Group I, (b) - Group III, IV and V compared between Group II.

1 units/mg protein required to inhibit 50% of adrenaline autooxidation,

2 µ moles of H_2_O_2_ consumed/min/mg protein,

3 µg of thio ester formed/min/mg protein,

4 µg of GSH/mg protein,

5 & 6 mg/g wet tissue.

### Effect of CP-P on reduced glutathione activity of non-enzymatic antioxidant

Non-enzymatic antioxidants are also involved in the tumor reduction after the treatment with CP-P (Tables 1). GSH plays a vital role in tissue repair. The combination of CP-P+CYC that was also influenced higher levels of GSH after 3 weeks treatment when compared to normal controls (group I). In group II, BCC rats treated with CP-P or CYC alone (after 20 days) exerted a similar effect on the enzyme levels like the untreated BCC rats. However, the CYC-treated rats showed severe toxicity at 0.2 mg/kg concentrations (group IV), as a result of which potential reduction in enzyme levels were found. The vitamin C content was drastically reduced after 90 days (13 weeks) in the BCC rats by the direct effect of DMBA during the carcinogenic processes. However, the CP-P alone treated rats (group III) had the most significantly elevated levels of vitamin C versus the combination of CP-P+CYC treated rats.

### Effect of CP-P on vitamin E (α-Tocopherol) levels

Vitamin E is a naturally occurring compound known as tocopherols and tocotrienols. In this study, the vitamin E levels were reduced tremendously by DMBA in untreated BCC rats (group II). Group III treated with CP-P (0.2 mg/kg) showed significant elevated levels of vitamin E. More interestingly, the combination (0.1 mg/kg of CP-P+CYC each) treated rats (group V) greatly increased the vitamin E levels versus BCC control. Additionally, CYC (0.2 mg/kg) treated groups (IV) also exerted the higher level of vitamin E content. There was a gradual reduction of vitamin content due to the toxic side effects by CYC following 20 days treatment.

## Discussion

In the present study, we investigated the effects of a natural protein product (CP-P) as a complementary therapy in DMBA-induced mammary tumorigenesis model. First, we found a significant *in vitro* cytotoxic activity of CP-P, with or without CYC, in breast cancer cell lines MCF-7 and MDA-MB-231. Second, we also found that CP-P induced significant apoptotic effects in MCF-7 cells, with or without CYC, which may account for its potential anti-cancer effects. Third, we also observed that CP-P suppressed NF-κB activation induced by TNF-α leading to the suppression of IκBα phosphorylation and degradation. We also observed that the suppression of TNF-α-induced IKK activation leads to the inhibition of IκBα phosphorylation and degradation in MCF-7 cells. CP-P also down-regulated the expression of various NF-κB-dependent gene products involved in cell proliferation (e.g. cyclin D1), and anti-apoptosis (e.g. Bcl-2). Several lines of evidence support the fact that NF-κB plays an essential role in the development and progression of breast cancer. First, studies have suggested the presence of constitutively active NF-κB at an early stage of neoplastic transformation of mammary cells [48]. Second, NF-κB activation can inhibit apoptosis in mouse mammary epithelia [49]. Third, selective activation of NF-κB subunits has been found in human breast cancer cell lines and in patient samples [50]. Fourth, inhibition of NF-κB in breast cancer cells can induce spontaneous apoptosis [51]. Also, it has been shown that the breast cancer tissue of women not responding to chemotherapy expresses constitutively-active NF-κB [52]. Thus, inhibition of NF-κB activation by CP-P may account for its anti-proliferative, pro-apoptotic and chemosensitizing properties as described here.

Additionally, we also observed the potential inhibitory effects of CP-P on the development of DMBA-induced breast cancer in rats. The 0.2 mg/kg dose of CP-P alone treated animals showed only 60.5% inhibition or reduction of tumor. A 0.2 mg/kg dose of CYC alone treated animals displayed at least 70% tumor reduction whereas the combination of CP-P (0.1 mg/kg)+CYC (0.1 mg/kg) treatment caused 74.5% reduction of tumor and effectively inhibits DMBA-induced tumors in rats. A single dose of DMBA induces mammary tumors in approximately 90 to 95% of female Sprague-Dawley (SD) rats [11], [53], [54]. In our study, all the SD rats developed breast carcinomas after 90 days (13 weeks) that was confirmed by histopathological examination. Our results corroborate in part with the previous report, in which an alkaloid obtained from *Solanum pseudocapsicum* (2.5 mg/kg doses) showed similar antitumor activity with increased survival time of tumor-bearing rats [55]. However, this treatment regimen caused significant reduction in the body weight due to toxicity, but no toxicity was observed among CP-P treatment in SD rats in our experiments.

Histopathological examination confirmed that the normal control rats displayed normal neoplasm, the CYC treated group showed low grade differentiation demonstrated by giant multinucleated cells, infiltration of tissues and necrotic effects due to CYC on rats as well as decreased cell density, infiltration, higher level of fibrosis, reduction of inflammation, faster neovascularization, non-invasiveness of tissue architecture observed in CP-P treated rats. However, the combination of CP-P+CYC treated group V showed glandular structures, an indication of functional differentiation due to maximal proliferative rates in the vasculature and epithelium as a result of antitumor effect. These data suggest that combination treatment prevents tumor progression significantly. As a result, the total number and volume of tumors per rat was decreased in the treated groups. In our previous study, pathological changes confirm the formation of tumor tubules and neovascularization after treatment [53].

Furthermore, the activities of enzymatic and non-enzymatic anti-oxidants were examined in the liver and mammary glands before and after 3 weeks treatment. CP-P treated groups (III, oral 0.2 mg/kg for 20 days) showed significantly (P<0.01*) elevated levels of SOD. The combination (CP-P+CYC)-treated group V exerted significant changes in the enzyme SOD levels than the CYC alone-treated rats. Glutathione S-transferases (GSTs) are a family of isoenzymes that play an important role in protecting cells from cytotoxins and carcinogens [56], [57]. In addition GSTs can bind and sequester intracellular toxins as well as prevent oxidative damage by an intrinsic organic peroxidase activity that converts toxic peroxidase to inactivated alcohols [58], [59]. In this study, we found that the CP-P treated group showed significantly higher levels of GSTs than the combination treated group. The GSH levels were significantly reduced in BCC control groups. The combination treatment (CP-P+CYC) also induced higher GSH levels 3 weeks following the treatment. However, the CYC treatment alone showed a significant increase in GSH levels than the BCC groups.

We also observed that in the BCC-group, the vitamin C content was drastically reduced after 90 days (13 weeks) due to carcinogen exposure. Whereas, in the CP-P administered group (III), a significant increase in vitamin C levels were noted, which may also account for its potential anti-cancer effects. Interestingly, it has been reported previously that the intake of vitamin C significantly reduces the incidence and growth of mammary tumors, and therefore has strong potential as a useful therapeutic for inhibiting breast cancer development [60].

Additionally, we also observed that vitamin E levels were significantly reduced by DMBA in BCC rats. Interestingly, CP-P treated rats also had increased vitamin E levels, which may also account for the observed anti-proliferative effects of CP-P. CP-P also increased the hepatic UDP-glucuronosyltransferase (UDP-GT) and glutathione-s-transferase (GST) activities. There was a decrease in the mammary DNA-DMBA adducts under the same treatment regimen that led to suppression of DMBA-induced mammary carcinogenesis. In addition, the combination treatment induced an apoptotic effect evidenced by TUNEL staining and significantly decreased the expression of cyclin D1 and Bcl-2 in breast tumor tissues.

## Conclusions

Results from this study confirm that CP-P has potential anticancer activity which is mainly attributed to its effect on the proliferation, apoptosis and redox status in breast cancer cells. Anticancer action occurs through the inhibition of the NF-κB activation cascade. Further studies are warranted to elucidate the anticancer potential of CP-P in breast cancer and use for other inflammatory conditions.

## Materials and Methods

### Chemicals and reagents

Phosphotungstic acid, thiobarbituric acid, trichloroacetic acid, acetic acid, sodium salicylate, and ethylenediaminetetraacetic acid were purchased from Hi-media Chemicals (Pvt) Ltd, Mumbai, India. Reduced Glutathione and 5, 5′Dithiobis (2-nitrobenzoic acid (DTNB), carcinogen 7, 12-Dimethylbenz(a)anthracene (DMBA), and β-actin antibody were purchased from Sigma Chemicals Co (St. Louis, MO, USA). Ascorbate, iron (III) chloride (FeCl_3_), sodium tungstate, sodium nitrate, methanol (Merck, Germany) and other reagents were of analytical grade. The drug cyclophosphamide (CYC) was obtained from the Apollo pharmacy, Chennai-600034. Antibodies against p65, p50, IkappaBalpha (IκBα), cyclin D1, B-cell lymphoma 2 (Bcl-2), glyceraldehyde 3-phosphate dehydrogenase (GAPDH), IkappaB kinase alpha (IKKα), and poly (ADP-ribose) polymerase (PARP) were obtained from Santa Cruz Biotechnology (Santa Cruz, CA, USA). Phospho-specific anti-IκBα (Ser32), phospho-specific anti-p65 (Ser536), and phospho-specific IKKα/β (Ser-180/181) were purchased from Cell Signaling (Beverly, MA, USA).

### Collection and identification

Root-bark of *C. procera* (CP) was collected in Thiruthani, Thiruvalluvar district, Tamil Nadu, India and properly identified [61]. A voucher specimen was prepared and stored at the institute. The fresh root-bark was separated, cut into small pieces by using a sterile razor blade and shade dried at 31°C (room temperature). This is a very commonly available medicinal plant and no specific permits were required for the described field studies. It is also confirmed that the location of the plant collection area is not privately-owned or protected. Additionally, field studies did not involve any endangered or protected plant species.

### Purification of antitumor protein

The root-bark extract was used for purifying antitumor protein. The dried material was powdered with an electric blender. Powdered material (50 g) was suspended in 250 ml of 50 mM Tris-hydrochloric acid buffer (pH 7.4), and the suspension was centrifuged at 250 g at 4°C for 15 min. Aliquots (5 ml) of the brownish clear supernatant were applied on a Superdex G-75 column (1.6×40 cm; Amersham Pharmacia, Sweden) previously equilibrated and eluted with the same buffer. Fractions (2 ml each) were collected at a flow rate of 12 ml/h. Absorbance of all fractions was monitored at 280 nm. Five fractions (CP-1, CP-2, CP-3, CP-4, and CP-5) were collected from the single pool of clear CP supernatant fractionated on the Superdex column, with aliquots taken for protein measurement. The CP-3 fraction with highest protein content and antitumor activity was further purified by reverse-phase high-performance liquid chromatography (C18, RP-HPLC) into two peaks (CP-F1 and CP-F2). The CP-F1 fraction was further separated by a C8 column (Jupitor Phenomex) in 0.1% aqueous trifluoroacetic acid (TFA) (Sigma Co, USA), and eluted with a linear gradient of 80% acetonitrile (ACN) in 0.1% TFA. These fractions were monitored at 215 nm [62], [63]. Total protein in the entire above fractions was estimated using the Bradford [64] reagent (Bio-Rad, Hercules, CA, USA) before immediate processing or storage at −80°C.

### Mass spectrometry analysis

Analyses were performed primarily using a perspective biosystem matrix-assisted laser desorption ionization-time of flight (MALDI-TOF/MS) Voyager-DE™ mass spectrometer operated in delayed extraction mode. The biologically active fraction was analyzed using a saturated solution of α-cyano-4-hydroxycinnamic acid (Sigma Co, St Louis, MO, USA) in acetone containing 1% TFA. The active fraction was selected in the mass range of 1–70 kDa. Spectra were calibrated using the sequazyme mass standards kit (Perspective Biosystems, Framingham, MA). The database search program for mass [MS-Fit], was used in National Center for Biotechnology Information (NCBI) and matrix science mascot was used in the matrix science-help-sequence database (MSDB). MALDI-TOF mass spectrometry was used for molecular mass determination.

### Cell line and cell culture

Human breast cancer cell lines MCF-7 and MDA-MB-231 were obtained from the American Type Culture Collection (Manassas, VA, USA). The cells were cultured under standard tissue culture conditions and tested negative for mycoplasma contamination. The cells were maintained in Roswell Park Memorial Institute (RPMI) 1640 medium (Gibco, Grand Island, NY, USA), supplemented with 10% w/v fetal bovine serum (FBS) (Gibco), 1 mM sodium pyruvate, penicillin (100 units/ml) and streptomycin (100 units/ml) (Gibco) in a 5% CO_2_ incubator at 37°C. Serum-free medium was used for the above experimental conditions. The cells were grown up to 80% confluence prior to an experiment.

### Assay for cytotoxicity

The antiproliferative effect of CP-P against MCF-7 and MDA-MB-231 cells was determined by the 3-(4, 5-Dimethylthiazol-2-yl)-2, 5-diphenyltetrazolium bromide (MTT, Sigma Chemicals Co, St. Louis, MO, USA) dye uptake method as described previously [65]. Briefly, cells (10^6^/ml) were incubated in triplicate in a 96-well plate with or without different concentrations of CP-P or CYC alone, or in combination (800–12.5 µg/ml) in a final volume of 0.2 ml for indicated time intervals at 37°C. Thereafter, MTT (20 µl of 5 mg/ml in PBS) was added to each well. After a 2 h incubation at 37°C, 0.1 ml lysis buffer (20% SDS, 50% dimethyl-formamide) was added; incubation was continued overnight at 37°C and optical density (OD) at 490 nm was measured by a BenchMark Plus plate reader (BioRad Laboratories, Hercules, CA, USA). The percent of cytotoxicity was calculated by comparing with the untreated control.

### Lactate dehydrogenase (LDH) release assay

The lactate dehydrogenase (LDH) release into media was an indicator of cell death detected according to the manufacturer's instructions (Roche, Indianapolis, USA). Briefly, after incubation, 200 µl of cell medium was transferred to a sterile 96-well plate with 50 µl of LDH reagent. After the reaction, the brownish red color development was measured at 440 nm by ELISA reader (Bench Markplus, Bio-Rad, CA, USA). Results are expressed as the percentage of LDH release versus control cells [66].

### Morphological changes of MCF-7 cells as determined by Transmission Electron Microscopy (TEM)

TEM of CP-P alone, or in a combination with CYC, treated MCF-7 cancer cells were performed as previously described [54]. Cells (2×10^5^ cells/ml) were seeded onto sterile cover glass. Cells were initially fixed in 2.5% glutaraldehyde followed by post-fixation with 1% osmium tetroxide. Samples were then dehydrated with an ascending series of ethyl alcohol baths (50–100%) before subsequent embedding in araldite resin. Semi- and ultra-thin sections were cut using a glass knife. The ultrathin sections were doubly stained with uranyl acetate and lead citrate before viewing under an Olympus EM2085 microscope (Philadelphia, PA, USA).

### Cell cycle analysis

The phases of cell cycle distribution following CP-P alone, or in combination with CYC, treatment of MCF-7 cells were analyzed after propidium iodide (PI) staining by flow cytometry [67]. Control and treated (IC_50_ dose, 24 h) MCF-7 cells were spun out of culture medium, washed with cold PBS, and fixed in 70% ethanol. The cells were then washed again, treated with DNase-free RNase A (10 µg/ml) at 37°C for 30 min, and stained with PI (200 µl from 50 µg/ml) for 15 min at 37°C. Cell cycle phase distribution of nuclear DNA was measured in a FACS caliber single laser flow cytometer (Becton Dickinson, Franklin Lakes, NJ, USA) using a 488 nm argon laser light source and 623 nm band pass filter using Cell Quest software (n = 4).

### Western blot analysis

To determine protein expression levels, we prepared extracts and fractionated them by SDS-polyacrylamide gel electrophoresis (SDS-PAGE) as described previously [68]. After electrophoresis, the proteins were electrotransferred to a nitrocellulose membrane, blocked with 5% nonfat milk to minimize non-specific binding, and probed with protein-specific antibodies (1∶3000) overnight at 4°C. Blots were washed, exposed to HRP-conjugated secondary antibodies for 2 h, and the expression of various proteins was detected by chemiluminescence emission (ECL; GE Healthcare, Little Chalfont, Buckinghamshire, UK).

### Terminal transferase dUTP nick end labeling (TUNEL) assay

Activation of endonucleases that cleave chromosomal deoxyribonucleic acid (DNA) is a hallmark of apoptosis. DNA fragmentation revealed by a multitude of DNA strand breaks, therefore, is considered the gold standard for identifying apoptotic cells. Several methodologies are based on fluorochrome-labeling of 3-OH termini of DNA strand breaks *in situ* with the use of exogenous terminal deoxynucleotidyl transfer (TdT). This is commonly defined as the TUNEL assay [69]. MCF-7 cells (10^6^ cells/well) were grown in a 96-well plate and exposed to CP-P or CYC alone or in combination, for 24 h. Cells without treatment served as controls. Cells were fixed with 4% paraformaldehyde for 10 min. After fixation at room temperature, cells were washed twice with PBS for 5 min each and then incubated with 3% hydrogen peroxide (H_2_O_2_) to block endogenous peroxidase activity. After 10 min, the cells were incubated in the labeling reaction mixture containing TdT at 37°C for 1 h. The reaction was terminated by adding stop buffer and cells were washed 3 times with PBS for 2 min each. Cell nuclei were counterstained with Hoechst 33342 (Sigma-Aldrich Co, St. Louis, MO, USA) and mounted upon a cover slip with Dako fluorescent mounting medium. Cells were observed under fluorescence microscope (Olympus Corporation, Center Valley, PA, USA).

### *In vivo* anticancer effect of CP-P in rats

Six week old female Sprague Dawely rats (250–300 gm body weight) were obtained from the National Institute of Nutrition, (Hyderabad-500 007, India) and divided into five groups (n = 5 per group), rats subsequently maintained in a controlled condition including 12 h light and 12 h dark cycles. Standard diet and tap water was ad libitum. The experiment was conducted according to the guidelines of the Institutional Animal Ethical Committee for Experimentations on Animals (IAECEA), and approved by the University of Madras, India. Group I served as a normal control animals (NC), group II - breast cancer bearing-animals treated with saline served as the breast cancer control (BCC), group III - breast cancer-bearing animals treated with CP-P alone (0.2 mg/kg, oral), group IV - breast cancer-bearing rats treated with CYC alone 0.2 mg/kg, i.p) and group V - breast cancer-bearing animals treated with CP-P (0.1 mg/kg, oral)+CYC (0.1 mg/kg, intraperitoneaļ i.p.). Induction of breast cancer: 1 mg of DMBA was dissolved in 0.5 ml of sunflower oil and 0.5 ml saline injected by subcutaneous injection beneath the mammary gland on either side (group II–V). The solid tumor developed after 90 days. After 13 weeks, the breast tumor-bearing rats were treated for 20 days [54], [70], [71].

### Histopathological examination of tumor tissues

Macroscopic mammary tumors, fixed in 10% buffered formalin, were embedded in paraffin using a conventional automated system. The blocks were cut to obtain 5 µm thick sections and stained with hemotoxylin-eosin (H & E) for histological examination [71]. Serial paraffin sections of each tissue image were captured by light microscopy (Olympus BX51, Philadelphia, USA).

### Breast tissue homogenate for biochemical analysis

The overnight-fasted animals were sacrificed by cervical decapitation; breast tissues were excised and quickly removed. The tissue (50 mg) was washed thoroughly with chilled PBS, cut into small pieces with a sterile heavy-duty blade, and finally homogenized on ice. The homogenate was centrifuged at 10,000 rpm for 15 min and supernatant diluted with PBS to a final concentration of protein (30 µg/ml). The biochemical anlysis of enzymatic antioxidant such as superoxide dismutase, catalase, glutathione-s-transferase, and non-enzymatic antioxidants such as reduced glutathione, vitamin C and E were estimated in the treated and untreated breast tissues of control rats.

### Superoxide dismutase (SOD) radical scavenging activity

Superoxide activity was assessed by the nitroblue tetrazolium (NBT) reduction method [72]. Test materials ranging from 1–150 mg/ml were added into reaction mixtures containing 0.1 mM EDTA (200 µl), 0.12 mM riboflavin (50 µl) and 0.6 M phosphate buffer (pH 7.8) in a final volume of 3 ml. Optical density was determined at 560 nm.

### Estimation of glutathione-s-transferase (GST) activity

Glutathione-S-transferase was assayed by the method described by Habig et al, [73]. The tissue samples were homogenized with 10 ml of 1-chloro-2, 4-dinitrobenzene (CDNB) phosphate buffer (0.5 M pH 6.5) and centrifuged at 12,000 rpm for 10 min. The supernatant was made up to 2.5 ml with double distilled water. The reaction mixture was pre-incubated at 37°C for 5 min. GSH (0.1 ml) was added and absorbance was measured at 340 nm for 3 min intervals. The enzyme activity was expressed as µg of thioester formed/min/mg protein of tissue.

### Determination of catalase (CAT) activity

The catalase activity was determined spectrophotometrically according to the method described by Johansson and Borg, [74]. Tissue (50 mg) was homogenized in 1.0 ml of phosphate buffer (50 mM, pH 7.0) and centrifuged at 10,000 rpm for 10 min. Hydrogen peroxide (H_2_O_2_) 0.4 ml was added to the supernatant. The reaction was stopped at 15, 30, 45 and 60 seconds by adding 0.2 ml of dichromate acetic acid. Tubes were boiled for 10 min, cooled and read at 620 nm. The standards for H_2_O_2_, ranging from 20–100 µm, were processed as above. Catalase activity was expressed as µmoles of H_2_O_2_ decomposed/min/mg of tissue protein.

### Estimation of reduced glutathione (GSH) activity

Glutathione peroxide was assayed by the method described by Forstrom et al, [75]. 0.2 ml each of EDTA (2.34 mg/ml of sodium azide), glutathione reduced (GSH) 3 mM (12.29 mg of GSH was dissolved in 10 ml of water. Hydrogen peroxide (2.5 mM, 0.085 ml of H_2_O_2_ was made up to 10 ml with double distilled water). Phosphate buffered solution (0.3 mM) and 50 mg of tissue homogenate or lysate were mixed and incubated at 37°C for 10 min. The reaction was arrested by adding 0.5 ml of trichloroacetic acid (TCA) (10%) and tubes were centrifuged 0.5 ml of supernatant. 0.3 ml of phosphate solution and 1.0 ml of DTNP-2,2′-dithiobis-(5-nitropyridine) (6 mM), 40 mg of DTNB dissolved in 100 ml of 1% trisodium citrate was added and colour development was read at 420 nm using a spectrophotometer. Glutathione activity was expressed as µg glutathione utilized/min/mg protein.

### Estimation of vitamin C (Ascorbic acid) levels

Ascorbic acid level was estimated by the method described by Omaye et al, [76]. Breast tissue homogenate 0.5 ml was mixed with 0.5 ml of distilled water and 0.1 ml of 5% TCA were added, mixed thoroughly and centrifuged at 1500 rpm for 20 min (4°C). Supernatant (1 ml) was mixed with 0.2 ml of dithiocarbonate (DTC) reagent and incubated at 37°C for 3 h. 1.5 ml of 65% sulphuric acid was added and mixed well. The solution was allowed to stand at room temperature for another 30 min, and absorbance read at 520 nm. The graded amount of standards was also carried out similarly. The ascorbic acid level was expressed as mg/g of fresh tissue.

### Estimation of vitamin E (α-Tocopherol)

Vitamin E level was determined by the method described by Gupta et al, [77] with slight modifications. Tumor tissues (100 mg) were weighed and homogenised with 1.5 ml of buffer solution. The tissue homogenate was put into 5 screw capped centrifuge tubes and centrifuged at 10,000 rpm for 15 min (4°C). 1.5 ml of xylene was then added to all tubes and shaken well. After centrifugation, 0.1 ml of the xylene layer was taken and 1.0 ml of dipyridyl reagent added, mixed well and read at 460 nm against the blank (1.5 ml distilled water) using a spectrophotometer. Afterwards, 0.33 ml of ferric chloride reagent was added to all tubes incubated for 90 min. The test and standards were read at 520 nm against the blank using a spectrophotometer.

### Statistical analysis

Statistical comparisons between control and treatment mean values of two parameters were analyzed using the Student's *t*-test. Multiple comparisons were done using ANOVA. The differences were statistically significant at *P<0.01; **P<0.05 levels.

## Supporting Information

File S1 Detailed Methodology.
(DOC)

Table S1 Detailed methodology for the supplementary materials of the amino acid sequence of the apolipoprotein A-I to which CP-P has been matched. A peptide summary report and clearer picture of MS/MS by mascot search results.
(DOC)

Table S2 Showed the highest score of apolipoprotein A-I matched with the CP-P protein.
(DOC)
