# Supplementary material for: Identification of a Novel Calotropis procera Protein That Can Suppress Tumor Growth in Breast Cancer through the Suppression of NF-κB Pathway
Source: PLoS One. 2012 Dec 20;7(12):e48514. doi: 10.1371/journal.pone.0048514 (PMC3527472; doi:10.1371/journal.pone.0048514)
Supplement: File S1 — Detailed Methodology. (DOC) [file pone.0048514.s001.doc]

**Detailed Methodology:**

***Extraction of Protein***

The protein was extracted from the root-bark of *C. procera* and dry powdered materials dissolved in 50 mM Tris-hydrochloric acid buffer (Tris-HCl, pH 7.4). The extract (suspension) samples were centrifuged at 250 g for 15 min (4°C), retained the supernatant and stored at -80°C for further analysis.

***Analysis of SDS-PAGE***

The final pure fraction of CP-P was performed by Sodium dodecyl sulphate polyacrylamide gel electrophoresis (SDS-PAGE) according to the method of Laemmli 1970. The separating gel (10%) and stacking gel (5%) polyacrylamide were used for SDS-PAGE. The CP-P dissolved in sample buffer and 20 µg of protein loaded into the well. After electrophoresis, the gel was fixed and stained with Coomassie brilliant blue R-250 for 24 h.

***Ingel Digestion***

From the SDS-PAGE, single protein band was excised, reduced, and alkylated prior to in-gel digestion with 2 μg (20 ng/μl) of modified porcine trypsin per protein spot. Extracted peptides were then desalted using Zip-Tip method (Agilent cleanup C18 Pipette Tips) according to the manufacturer’s instructions.

***MALDI-TOF Mass***

The trypsin eluted protein was analyzed by MALDI-TOF Voyager-DE^TM^ mass spectrometer (Biosystem, Framingham, MA) and performed with 1 µl of digested peptide and a saturated solution of α-cyano-4-hydroxy cinnamic acid, CHCA (Sigma Co, St Louis, MO, USA) in 50% Acetonitrile and 0.1% Trifluoro acetic acid.

***MS/MS data Analysis***

The resultant mass spectra and its sequence data were analyzed automatically by database matching against the National Center for Biotechnology Information (NCBInr, Bethesda, USA) protein database, using the Mascot data were searched against the NCBI database using Mascot database search engine (Matrix Science, London, UK; [http://www.matrixscience.com](http://www.matrixscience.com/)) program (**Table S1-S2)**.
