# Supplementary material for: Identification of a Novel Calotropis procera Protein That Can Suppress Tumor Growth in Breast Cancer through the Suppression of NF-κB Pathway
Source: PLoS One. 2012 Dec 20;7(12):e48514. doi: 10.1371/journal.pone.0048514 (PMC3527472; doi:10.1371/journal.pone.0048514)
Supplement: Table S1 — Detailed methodology for the supplementary materials of the amino acid sequence of the apolipoprotein A-I to which CP-P has been matched. A peptide summary report and clearer picture of MS/MS by mascot search results. (DOC) [file pone.0048514.s002.doc]

**Table S1.** Detailed methodology for the supplementary materials of the amino acid sequence of the apolipoprotein A-I to which CP-P has been matched. A peptide summary report and clearer picture of MS/MS by mascot search results.

|  | **Accession** | **Mass** | **Score** | **Description** |
| --- | --- | --- | --- | --- |
| **1.** | **[gi|109571](../../../../C:%5CDocuments%20and%20Settings%5CMICRAMA%5CLocal%20Settings%5CTemp%5CTemporary%20Directory%2015%20for%202D-ms%20result%202.zip%5C2nd%5C16%5CE2.html#Hit1%23Hit1)** | **30358** | **290** | **apolipoprotein A-I precursor - mouse** |
| **2.** | **[gi|26345182](../../../../C:%5CDocuments%20and%20Settings%5CMICRAMA%5CLocal%20Settings%5CTemp%5CTemporary%20Directory%2015%20for%202D-ms%20result%202.zip%5C2nd%5C16%5CE2.html#Hit2%23Hit2)** | 30597 | 290 | unnamed protein product [Mus musculus] |
| **3.** | **[gi|6753096](../../../../C:%5CDocuments%20and%20Settings%5CMICRAMA%5CLocal%20Settings%5CTemp%5CTemporary%20Directory%2015%20for%202D-ms%20result%202.zip%5C2nd%5C16%5CE2.html#Hit3%23Hit3)** | 30569 | 289 | apolipoprotein A-I [Mus musculus] |
| **4.** | **[gi|74203337](../../../../C:%5CDocuments%20and%20Settings%5CMICRAMA%5CLocal%20Settings%5CTemp%5CTemporary%20Directory%2015%20for%202D-ms%20result%202.zip%5C2nd%5C16%5CE2.html#Hit4%23Hit4)** | 30666 | 288 | unnamed protein product [Mus musculus] |
| **5.** | **[gi|148693731](../../../../C:%5CDocuments%20and%20Settings%5CMICRAMA%5CLocal%20Settings%5CTemp%5CTemporary%20Directory%2015%20for%202D-ms%20result%202.zip%5C2nd%5C16%5CE2.html#Hit5%23Hit5)** | 28517 | 287 | apolipoprotein A-I, isoform CRA_b [Mus musculus] |
| **6.** | **[gi|61402210](../../../../C:%5CDocuments%20and%20Settings%5CMICRAMA%5CLocal%20Settings%5CTemp%5CTemporary%20Directory%2015%20for%202D-ms%20result%202.zip%5C2nd%5C16%5CE2.html#Hit6%23Hit6)** | 23008 | 270 | Apoa1 protein [Mus musculus] |
| **7.** | **[gi|49259463](../../../../C:%5CDocuments%20and%20Settings%5CMICRAMA%5CLocal%20Settings%5CTemp%5CTemporary%20Directory%2015%20for%202D-ms%20result%202.zip%5C2nd%5C16%5CE2.html#Hit7%23Hit7)** | 24003 | 262 | Chain T, Trypsin Inhibitor In Complex With Bovine Trypsin Variant X(Sswi)bt.B4 |
| **8.** | **[gi|230338](../../../../C:%5CDocuments%20and%20Settings%5CMICRAMA%5CLocal%20Settings%5CTemp%5CTemporary%20Directory%2015%20for%202D-ms%20result%202.zip%5C2nd%5C16%5CE2.html#Hit8%23Hit8)** | 23975 | 262 | Chain E, Trypsin (E.C.3.4.21.4) Complex With Bowman-Birk Inhibitor (AB-I) |
| **9.** | **[gi|9955040](../../../../C:%5CDocuments%20and%20Settings%5CMICRAMA%5CLocal%20Settings%5CTemp%5CTemporary%20Directory%2015%20for%202D-ms%20result%202.zip%5C2nd%5C16%5CE2.html#Hit9%23Hit9)** | 23972 | 262 | Chain A, Recruiting Zinc To Mediate Potent, Specific Inhibition Of Serine Proteases |
| **10.** | **[gi|2392803](../../../../C:%5CDocuments%20and%20Settings%5CMICRAMA%5CLocal%20Settings%5CTemp%5CTemporary%20Directory%2015%20for%202D-ms%20result%202.zip%5C2nd%5C16%5CE2.html#Hit10%23Hit10)** | 23999 | 262 | Chain , Structure Of Hydrolase (Serine Proteinase) |
| **11.** | **[gi|2392548](../../../../C:%5CDocuments%20and%20Settings%5CMICRAMA%5CLocal%20Settings%5CTemp%5CTemporary%20Directory%2015%20for%202D-ms%20result%202.zip%5C2nd%5C16%5CE2.html#Hit11%23Hit11)** | 23961 | 262 | Chain A, Bovine Trypsin Complexed To Appi |
| **12.** | **[gi|49259461](../../../../C:%5CDocuments%20and%20Settings%5CMICRAMA%5CLocal%20Settings%5CTemp%5CTemporary%20Directory%2015%20for%202D-ms%20result%202.zip%5C2nd%5C16%5CE2.html#Hit12%23Hit12)** | 23980 | 262 | Chain T, Trypsin Inhibitor In Complex With Bovine Trypsin Variant X(Ssyi)bt.B4 |
| **13.** | **[gi|88193016](../../../../C:%5CDocuments%20and%20Settings%5CMICRAMA%5CLocal%20Settings%5CTemp%5CTemporary%20Directory%2015%20for%202D-ms%20result%202.zip%5C2nd%5C16%5CE2.html#Hit13%23Hit13)** | 23972 | 262 | Chain E, Crystal Structure Of A Bpti Variant (Cys14->ser) In Complex With Trypsin |
| **14.** | **[gi|88193018](../../../../C:%5CDocuments%20and%20Settings%5CMICRAMA%5CLocal%20Settings%5CTemp%5CTemporary%20Directory%2015%20for%202D-ms%20result%202.zip%5C2nd%5C16%5CE2.html#Hit14%23Hit14)** | 24087 | 262 | Chain E, Crystal Structure Of A Bpti Variant (Cys38->ser) In Complex With Trypsin |
| **15.** | **[gi|13096612](../../../../C:%5CDocuments%20and%20Settings%5CMICRAMA%5CLocal%20Settings%5CTemp%5CTemporary%20Directory%2015%20for%202D-ms%20result%202.zip%5C2nd%5C16%5CE2.html#Hit15%23Hit15)** | 24563 | 262 | Chain A, Bovine Beta-Trypsin Bound To Meta-Amidino Schiff Base Magnesium(Ii) Chelate |
| **16.** | **[gi|230347](../../../../C:%5CDocuments%20and%20Settings%5CMICRAMA%5CLocal%20Settings%5CTemp%5CTemporary%20Directory%2015%20for%202D-ms%20result%202.zip%5C2nd%5C16%5CE2.html#Hit16%23Hit16)** | 24662 | 262 | Chain , Trypsinogen (0.50 Methanol, 0.50 Water) |
| **17.** | **[gi|1421532](../../../../C:%5CDocuments%20and%20Settings%5CMICRAMA%5CLocal%20Settings%5CTemp%5CTemporary%20Directory%2015%20for%202D-ms%20result%202.zip%5C2nd%5C16%5CE2.html#Hit17%23Hit17)** | 24659 | 262 | Chain , Trypsinogen-Ca From Peg |
| **18.** | **[gi|5542503](../../../../C:%5CDocuments%20and%20Settings%5CMICRAMA%5CLocal%20Settings%5CTemp%5CTemporary%20Directory%2015%20for%202D-ms%20result%202.zip%5C2nd%5C16%5CE2.html#Hit18%23Hit18)** | 25388 | 261 | Chain A, Trypsin Inhibitors With Rigid Tripeptidyl Aldehydes |
| **19.** | **[gi|2507249](../../../../C:%5CDocuments%20and%20Settings%5CMICRAMA%5CLocal%20Settings%5CTemp%5CTemporary%20Directory%2015%20for%202D-ms%20result%202.zip%5C2nd%5C16%5CE2.html#Hit19%23Hit19)** | 26093 | 261 | Cationic trypsin precursor (Beta-trypsin) [Contains: Alpha-trypsin chain 1, 2] |
| **20.** | **[gi|34810822](../../../../C:%5CDocuments%20and%20Settings%5CMICRAMA%5CLocal%20Settings%5CTemp%5CTemporary%20Directory%2015%20for%202D-ms%20result%202.zip%5C2nd%5C16%5CE2.html#Hit20%23Hit20)** | 26077 | 261 | Chain B, Non-Covalent Complex Between Alpha-1-Pi-Pittsburgh And S195a Trypsin |
