# Supplementary material for: Identification of a Novel Calotropis procera Protein That Can Suppress Tumor Growth in Breast Cancer through the Suppression of NF-κB Pathway
Source: PLoS One. 2012 Dec 20;7(12):e48514. doi: 10.1371/journal.pone.0048514 (PMC3527472; doi:10.1371/journal.pone.0048514)
Supplement: Table S2 — Showed the highest score of apolipoprotein A-I matched with the CP-P protein. (DOC) [file pone.0048514.s003.doc]

**Table S2.** Showed the highest score of apolipoprotein A-I matched with the CP-P protein.

| **1.** | [gi|109571](../../../../C:%5CDocuments%20and%20Settings%5CMICRAMA%5CLocal%20Settings%5CTemp%5CTemporary%20Directory%2013%20for%202D-ms%20result%202.zip%5C2nd%5C16%5Cgi+109571.html)     **Mass:** 30358    **Score:** 290    **Expect:** 5.4e-023  **Queries matched:** 20 |
| --- | --- |

|  | **Observed** | **Mr(expt)** | **Mr(calc)** | **Delta** | **Start** |  | **End** | **Miss** | **Ions** | **Peptide** |
| --- | --- | --- | --- | --- | --- | --- | --- | --- | --- | --- |
|  | 1040.60 | 1039.59 | 1039.58 | 0.01 | 228 | - | 236 | 0 | --- | K.ARPALEDLR.H |
|  | 1047.57 | 1046.56 | 1046.54 | 0.02 | 164 | - | 172 | 0 | --- | R.LSPVAEEFR.D |
|  | 1237.64 | 1236.64 | 1236.61 | 0.02 | 131 | - | 139 | 1 | 44 | K.WKEDVELYR.Q |
|  | 1237.64 | 1236.64 | 1236.61 | 0.02 | 131 | - | 139 | 1 | --- | K.WKEDVELYR.Q |
|  | 1240.64 | 1239.63 | 1239.61 | 0.02 | 36 | - | 46 | 0 | --- | K.DFANVYVDAVK.D |
|  | 1266.66 | 1265.65 | 1265.63 | 0.02 | 120 | - | 129 | 0 | --- | K.VQPYLDEFQK.K |
|  | 1297.66 | 1296.65 | 1296.62 | 0.02 | 184 | - | 194 | 0 | --- | R.TQLAPHSEQMR.E |
|  | 1313.65 | 1312.64 | 1312.62 | 0.02 | 184 | - | 194 | 0 | --- | R.TQLAPHSEQMR.E + Oxidation (M) |
|  | 1318.70 | 1317.69 | 1317.67 | 0.02 | 164 | - | 174 | 1 | 19 | R.LSPVAEEFRDR.M |
|  | 1318.70 | 1317.69 | 1317.67 | 0.02 | 164 | - | 174 | 1 | --- | R.LSPVAEEFRDR.M |
|  | 1331.66 | 1330.65 | 1330.66 | -0.01 | 237 | - | 247 | 0 | --- | R.HSLMPMLETLK.T + 2 Oxidation (M) |
|  | 1340.75 | 1339.74 | 1339.71 | 0.03 | 142 | - | 154 | 0 | 64 | K.VAPLGAELQESAR.Q |
|  | 1340.75 | 1339.74 | 1339.71 | 0.03 | 142 | - | 154 | 0 | --- | K.VAPLGAELQESAR.Q |
|  | 1394.76 | 1393.76 | 1393.72 | 0.03 | 120 | - | 130 | 1 | 47 | K.VQPYLDEFQKK.W |
|  | 1394.76 | 1393.76 | 1393.72 | 0.03 | 120 | - | 130 | 1 | --- | K.VQPYLDEFQKK.W |
|  | 1522.78 | 1521.78 | 1521.78 | -0.01 | 118 | - | 129 | 1 | --- | K.QKVQPYLDEFQK.K |
|  | 1596.90 | 1595.89 | 1595.86 | 0.02 | 142 | - | 156 | 1 | --- | K.VAPLGAELQESARQK.L |
|  | 1655.83 | 1654.82 | 1654.80 | 0.03 | 36 | - | 50 | 1 | --- | K.DFANVYVDAVKDSGR.D |
|  | 1852.89 | 1851.88 | 1851.84 | 0.04 | 93 | - | 106 | 1 | --- | R.DFWDNLEKETDWVR.Q |
|  | 1998.00 | 1996.99 | 1996.97 | 0.02 | 184 | - | 200 | 1 | --- | R.TQLAPHSEQMRESLAQR.L + Oxidation (M) |

|  | **No match to:** 1020.53, 1082.57, 1111.58, 1167.56, 1220.61, 1300.66, 1359.68, 1365.72, 1405.68, 1433.75, 1489.71, 1490.76, 1493.74, 1567.74, 1638.88, 1639.91, 1868.89, 1882.97, 1884.90, 2045.12, 2145.09, 2145.09, 2161.02, 2163.09, 2163.09, 2185.07, 2193.02, 2194.04, 2210.01, 2217.01, 2226.03, 2251.05, 2256.13, 2273.20, 2273.20, 2289.19, 2289.19, 2295.13, 2305.09, 2306.12, 2328.06, 2344.04, 2404.29, 2501.30, 2549.27, 2550.27, 2550.27, 2581.30, 2612.23, 2706.33, 3136.46, 3153.47, 3154.49, 3211.53, 3211.53 |
| --- | --- |
